# Polyphosphate Ester-Type Transporters Improve Antimicrobial Properties of Oxytetracycline

**DOI:** 10.3390/antibiotics12030616

**Published:** 2023-03-20

**Authors:** Mariya Kozak, Anna Stasiuk, Vasyl Vlizlo, Dmytro Ostapiv, Yulia Bodnar, Nataliia Kuz’mina, Natalia Figurka, Natalia Nosova, Roman Ostapiv, Igor Kotsumbas, Sergiy Varvarenko, Volodymyr Samaryk

**Affiliations:** 1 Institute of Animal Biology of the NAAS (National Academy of Agrarian Sciences) of Ukraine, 79034 Lviv, Ukraine; oddost@ukr.net (D.O.);; 2 Department of Organic Chemistry, Lviv Polytechnic National University, 79000 Lviv, Ukraine; anna.v.stasiuk@lpnu.ua (A.S.);; 3 Department of Internal Animal Diseases and Clinical Diagnostics, S. Gzhytskyi National University of Veterinary Medicine and Biotechnologies Lviv, 79010 Lviv, Ukraine; vasyl.vlizlo@scivp.lviv.ua; 4 State Research Control Institute of Veterinary Medicinal Products and Feed Additives, 79019 Lviv, Ukraine

**Keywords:** oxytetracycline, poly(phosphoester)s, oxytetracycline-polyphosphate ester-type transporter complex, antibiotic, microorganisms

## Abstract

Prolonged use of antibiotics can cause toxicity in human and animal cells and lead to the development of antibiotic resistance. The development of drug delivery systems for enhanced antibacterial properties of antibiotics could reduce toxic effects and minimize the development of resistance. The aim of this study was to evaluate the effectiveness of oxytetracycline in complexes with new polyphosphate ester-type transporters and to investigate the antimicrobial effect of these complexes on *Escherichia coli*, *Pseudomonas aeruginosa*, and *Staphylococcus aureus* growth in vitro. Two polyphosphate ester-type transporters with different molecular weights were synthesized, and oxytetracycline was attached through the phosphorus groups. To determine the sensitivities of microorganisms, oxytetracycline hydrochloride and oxytetracycline complexes with polyphosphate ester-type transporters (P4 and P6) were added to liquid and solid media with *E. coli*, *P. aeruginosa*, and *S. aureus* in different doses. Oxytetracycline in complex with polyphosphate ester-type transporters at low doses (2.3 to 3.8 μg/disk or μg/mL) in both solid and liquid media inhibits the growth of *S. aureus* more effectively than oxytetracycline alone. The maximum influence on *E. coli* growth on solid media is observed at a dose of 8 μg/disk of oxytetracycline in combination with both P4 and P6 polyphosphate ester-type transporters. *P. aeruginosa* growth under the influence of oxytetracycline in combination with polyphosphate-ester type transporters in a liquid medium depends on the dose of antibiotic and the day of cultivation.

## 1. Introduction

The discovery of antibiotics was one of the greatest achievements in human history. Antibiotics transformed medicine and have saved many lives [1]. Unfortunately, the overuse of antibiotics, including oxytetracycline, led to the development of antibiotic resistance in bacteria [2,3,4]. Oxytetracycline was approved by the U.S. Food and Drug Administration (FDA) in 1950 [5]. Since that time, the emergence of tetracycline-resistant mechanisms has limited its use [5]. Efflux, ribosomal protection, and enzymatic inactivation of tetracyclines are known mechanisms of resistance to oxytetracycline [5,6,7]. Hospital-associated infections with antibiotic-resistant *Escherichia coli*, *Pseudomonas aeruginosa*, and *Staphylococcus aureus* have increased morbidity and mortality [1]. In addition to antibiotic resistance, *P. aeruginosa* is able to form biofilms [8], which consist of exopolysaccharides, extracellular DNA, proteins, and lipids [9]. The treatment of such infections is challenging and depends on antibiotics [10] and hydrolyzing glycosidase enzymes for efficient biofilm dispersal [9,11,12]. Prolonged use of antibiotics also causes toxicity to human and animal cells [13,14,15]. Oxytetracycline given intravenously in high doses is potentially nephrotoxic and may increase the risk of acute renal failure [16]. Adverse effects on the musculoskeletal and urinary systems of healthy foals have been associated with previously administered doses of this antibiotic [17]. The solution to antibiotic resistance and toxicity is achieved in two ways: by reducing the administered dose and by “targeted delivery”. Targeted delivery is achieved when the antibiotics are delivered directly to the organism using special molecules or carriers [18,19]. Effective targeted drug delivery systems developed in recent years include nanoscale biocompatible polymeric transport systems that penetrate bacterial membranes [20]. Antibiotics, combined with such carriers, can provide desirable therapeutic effects with reduced toxicity for both human and animal cells. We hypothesize that polymeric transport systems, specifically polyphosphate ester-type transporters, will improve the effectiveness of oxytetracycline. In this study, polymers of pseudo-polyamino acids were synthesized. These molecules combine the advantages of polyether diols (polyethylene glycol, polypropylene glycol), as components of polymeric surfactants for drug delivery, and the unique properties of amino acids: biocompatibility and non-toxic biodegradation [21]. In addition, these polymers meet all modern requirements for polymer conveyors. Unlike polymers of pseudo-polyamino acids, polyphosphate esters contain a phosphorus group for attaching oxytetracycline. It has also been shown that glutamic acid-based polyphosphoesters have surfactant properties and form various micellar associates in aqueous solutions, which can transport therapeutic agents in the body [22]. The aim of this study was to investigate the effectiveness of complexes between new polyphosphate ester-type transporters and oxytetracycline and to evaluate the effect of newly developed antimicrobial drug carriers on *E. coli*, *P. aeruginosa*, and *S. aureus* growth in vitro.

## 2. Materials and Methods

### 2.1. Synthesis of the Oxytetracycline Complex with a Polyphosphate Ester Type Transporter

The synthesis of the oxytetracycline complex with a polyphosphate ester type transporter was performed in two stages. In the first stage, polyphosphate esters [phosphorus-containing pseudo-poly(amino acid(s)] were obtained by activated polycondensation, according to the Steglich reaction of *N*-derivatives of dicarboxylic α-amino acids and di-polyethylene glycol (ethyl) phosphates [23]. PEG-400 or PEG-600 was used in the synthesis process of di-polyethylene glycol (ethyl) phosphates, yielding two kinds of polyesters with different molecular weights. In the second stage, oxytetracycline was attached to the polyphosphate esters through the phosphorus group. As a result, two compounds were synthesized: oxytetracycline + P4 and oxytetracycline + P6.

Phosphorus-containing polyesters (PPE) were synthesized according to the following method [24]. N-steroil-L-glutamic acid (GluSt) (5.17 g, 12.5 mmol), dipoly(ethylene glycol) ethyl phosphate (DEP) (9.97 g, 11.3 mmol), and dichloromethane were loaded into a reactor. A solution of DCC (5.67 g, 27.5 mmol) and the catalyst—DMAP (0.19 g, 1.6 mmol) were dripped into the reaction mixture at a temperature of 280 K. Then the reaction mixture was maintained at 288 K for 3 h and at 398 K for 3 h. Finally, a side product of the reaction, dicyclohexylurea (DCU), was filtered off and dichloromethane was evaporated. The polymer was purified from unreacted monomers, an activator, and a catalyst by precipitation in acetone from hexane. The product was dried under vacuum to a constant weight. Then the hydrolysis of ethyl phosphate group was performed in acidic conditions.

The molecular weight of PPE was determined by SEC and ^1^H NMR. In this work, polyesters with a molecular weight in the range of 3000 (P4-1300-one link)–6000(P6-1800-one link) g/mol were obtained. Polydispersity coefficient of polyesters within 1.2 ÷ 1.4.

Number-average molecular weight (Mn) and dispersity (Ð) were measured by size exclusion chromatography (SEC) in THF as eluent (flow 1 mL/min), at 35 °C, on a Waters^®^ chain 2707 autosampler equipped with a 1515 Isocratic Pump and a guard column (Styragel 30 × 4.6 mm) connected to a column (Styragel HR2 + HR4, 300 × 7.8 nm). The Waters^®^ 2996 PDA and Waters^®^ 2414 Refractive Index Detector were used. Calibration was performed with polystyrene (PS) standards ranging from 580 g/mol to 483,000 g/mol.

Nuclear magnetic resonance (NMR) analysis ^1^H NMR spectra were recorded at 400 MHz on a Bruker^®^ spectrometer. The samples were dissolved in chloroform-d (CDCl3). The chemical shifts (δ) are expressed in parts per million (ppm) relative to Me4Si and the coupling constants (J) in Hertz (Figure S1).

**Figure 1:**
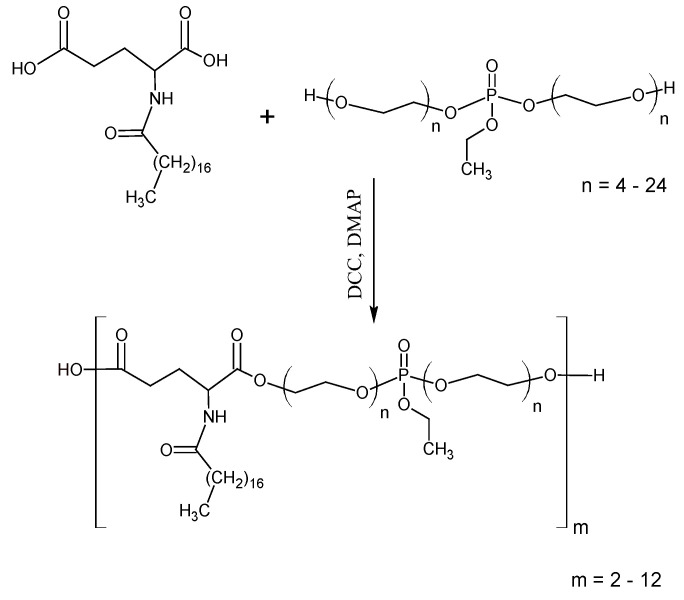
Synthesis of phosphorus-containing polyesters (PPE) with an ethyl phosphate group.

The phosphorus-containing polyesters (PPE) were synthesized by the Steglich reaction according to the scheme of Figure 1. In this study, N-stearoyl-glutamic acid was used as it gave high yields of PPAA of the polyester type described above. The ratio between the number of hydroxyl and carboxyl groups 9:10 provides an excess of hydroxyl groups. DCC was used in 10% excess to carboxyl groups.

### 2.2. Chromatography

The concentration of oxytetracycline in the products was measured by high-performance liquid chromatography using a diode array detector. The samples were separated on a Waters Luna C18 chromatograph 250 × 4.6 mm; 5 μm was used as a chromatographic column. The mobile phase was a mixture of acetonitrile and 0.2% phosphoric acid in a volume ratio of 2:8. Oxytetracycline was detected at 350 nm. The flow rate of the mobile phase was 1 mL/min, and the injection volume was 10 μL.

Oxytetracycline hydrochloride, manufactured by Sigma-Aldrich (St. Louis, MI, USA), with a sample purity of 98.7%, was used as a standard. Standard and test samples were dissolved to a concentration of 100 μg/mL.

### 2.3. Oxytetracycline Susceptibility Testing

Studies evaluating the effectiveness of the newly developed oxytetracycline complexes with polyphosphate ester-type P4 and P6 transporters were performed using serial dilutions in broth and agar, following previously described guidelines [25].

*S. aureus*, *P. aeruginosa*, and *E. coli* were cultured in liquid and solid media [meat-peptone broth with glucose 4% (Pharmreactive LLC, Ukraine)]. For inhibition assays, 2 mL (1.5 × 10^8^ CFU/mL) of the overnight culture was added to 20 mL of medium (at a temperature of 37 °C) on each plate. For growth inhibition experiments in liquid culture, 20 μL of overnight culture was added to 5 mL of liquid media in culture tubes. To prepare the overnight cultures, the microorganisms were transferred to tubes by loop inoculation with 10 mL of liquid nutrient medium and incubated for 24 h at 32 ± 2 °C.

To determine the sensitivities of microorganisms to the antibiotic on solid media, oxytetracycline hydrochloride and its complexes with polyphosphate ester-type (P4 and P6) were applied to paper disks (d = 10 mm) in the following doses: 2.3, 2.9, 3.8, and 5.4 μg/disk for *S. aureus*; and 8, 11, 14, and 20 μg/disk for *E. coli* and *P. aeruginosa* as previously described [26]. All experiments were performed in seven repetitions. TotalLab TL120 Software (Gosforth, UK) was used to measure the zones of inhibition in sm^2^ using camera images of disk diffusion plates.

### 2.4. Oxytetracycline Susceptibility Testing in Liquid Medium

To test the effectiveness of the transporters in liquid culture, 20 μL of inoculum of microorganisms (*S. aureus*, *E. coli*, and *P. aeruginosa*) were added to 5 mL of medium containing oxytetracycline hydrochloride (Sigma-Aldrich) or oxytetracycline polyphosphate ester complexes (P4 and P6) in doses: 2.3, 2.9, 3.8, and 5.4 μg/mL for all microorganisms and incubated at 32 °C for 24, 48, and 72 h. Culture growth in the presence of oxytetracycline or its complexes was compared to the negative control containing no antibiotic, which was stored at 2 to 4 °C. The absorption of the samples was measured spectrophotometrically in a cuvette (layer thickness of 10 mm) at a wavelength of λ = 660 nm (one timepoint/24 h). All experiments were repeated seven times.

## 3. Results

### 3.1. The Structure of the Polyphosphate Ester

**Figure 2:**
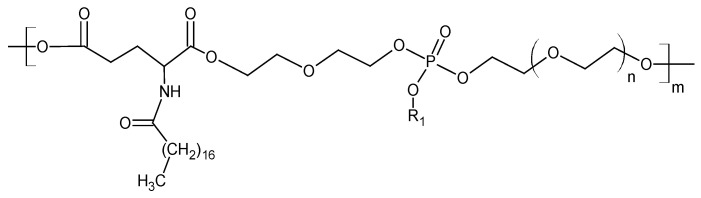
The structural formula of the polyphosphate ester.

The structural formula of the polyphosphate ester is represented by the general formula found in Figure 2. The obtained molecule contains lipophilic and hydrophilic fragments, which exhibit amphiphilic properties. It has surfactant properties and the ability to form self-stabilized dispersions with a nanometric dispersed phase in aqueous and physiological solutions [25,26]. The presence of a phosphate group (in the hydrophilic fragment after hydrolysis of the ethoxy group) provides conjugates with molecules that exhibit base properties [19]. Two polyphosphate ester transporters (P4 and P6) were synthesized.

### 3.2. Chromatography Studies

**Figure 3:**
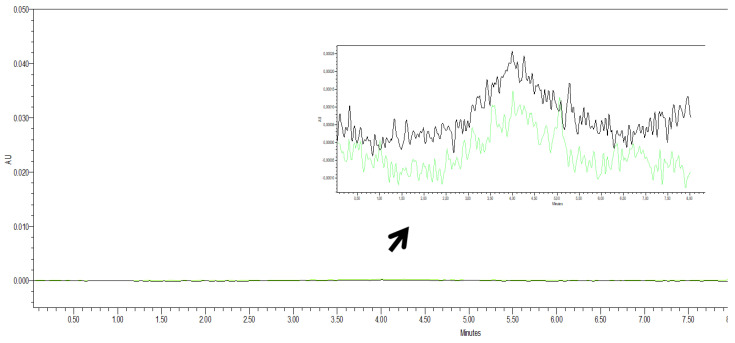
Chromatograms of polyphosphate ester-type transporters: P4 and P6. Note: P4 (green), P6 (black).

P4 and P6 transporters were tested by high-performance liquid chromatography. Only slight differences between the transporters were spotted in the chromatograms (Figure 3). The result showed that the polyphosphate carrier does not interfere with oxytetracycline detection and quantification using the high-performance liquid chromatography method.

**Figure 4:**
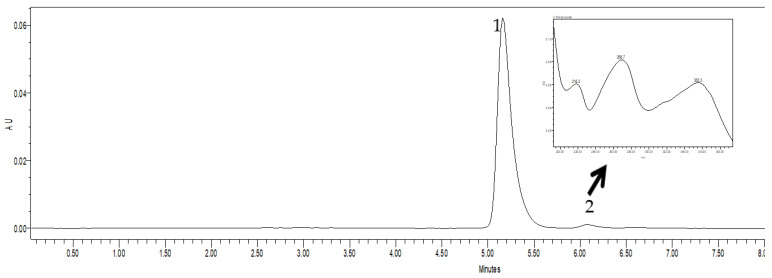
Chromatogram of a standard solution of oxytetracycline hydrochloride. Note: in this and the following figures: 1—the main peak of oxytetracycline; 2—concomitant components of the synthesis of the active substance.

**Figure 5:**
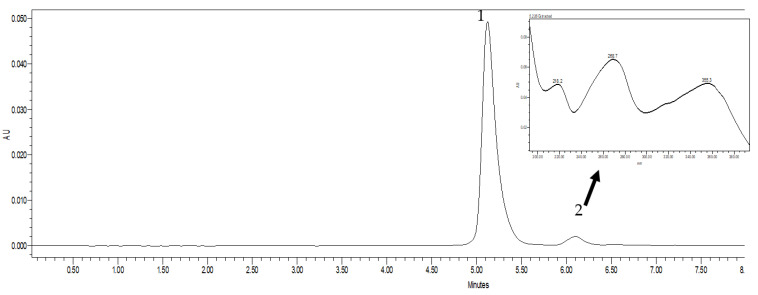
Chromatogram of oxytetracycline in the polyphosphate ester-type P4 transporter.

**Figure 6:**
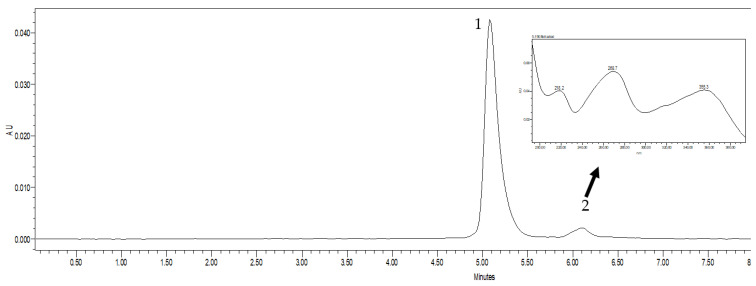
Chromatogram of oxytetracycline in the polyphosphate ester-type P6 transporter.

Comparative analysis of the chromatograms of the standard solution of oxytetracycline hydrochloride (Figure 4) and its complexes in the composition of polyphosphate ester-type transporters P4 (Figure 5) and P6 (Figure 6) did not represent significant differences.

Along with the main peak of oxytetracycline, there are three peaks with reduced mobility in the chromatograms. Under these conditions, an antibiotic base and accompanying unidentified components are detected. The proportion of unidentified components ranges from 1.4 to 4.7%. Oxytetracycline content was 11.9 mg/mL in the oxytetracycline + P4 complex and 12.2 mg/mL in the oxytetracycline + P6 complex. The results show that the efficiency of complex formation between polyphosphate ester-type transporters (P4 and P6) and oxytetracycline is similar for the two studied polymers.

### 3.3. Antibacterial Activity

**Figure 7:**
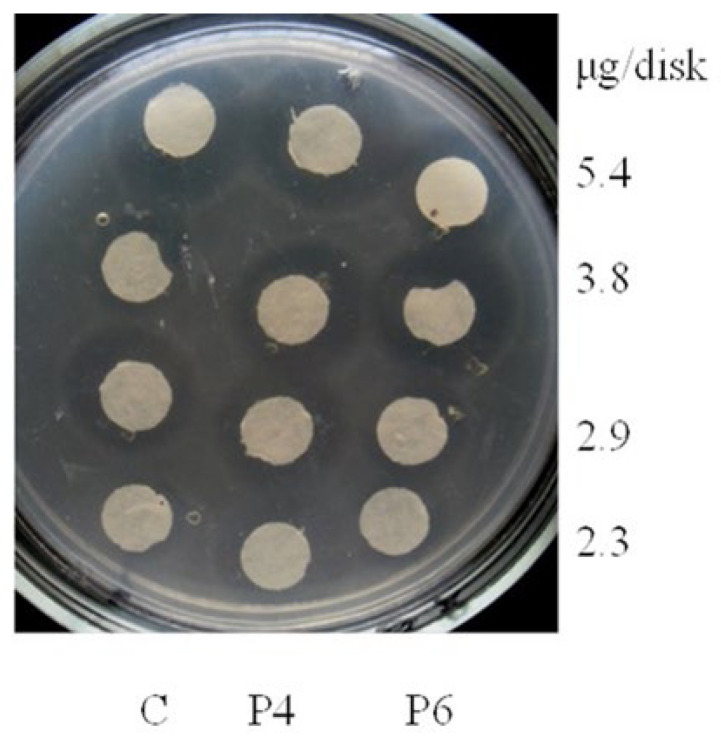
Growth inhibition of *Staphylococcus aureus* (*S. aureus*). In this and the following figures: C—control (oxytetracycline hydrochloride); P4 and P6—oxytetracycline in complex with polyphosphate ester-type transporters.

Oxytetracycline, when complexed with polyphosphate ester transporters, increased the susceptibility of *S. aureus* to oxytetracycline. Inhibition of *S. aureus* growth is most pronounced at the minimum dose (2.3 μg/disk) of oxytetracycline + P4 (Figure 7, Table 1). The growth inhibition is higher by 37.5% (*p* < 0.01) compared to the actions of oxytetracycline hydrochloride and oxytetracycline + P6. The growth inhibition of the microorganism was observed using higher doses of oxytetracycline + P4. The inhibition of growth was also greater compared to controls (*p* < 0.01).

Oxytetracycline + P6 is more effective on *S. aureus* at 3.8 μg/disc compared to the control. The correlation ratio of the influence of oxytetracycline hydrochloride and in complexes with polyphosphate ester-type transporters on the growth inhibition zone of *S. aureus* depends on the dose of antibiotic and is strong (η^2^ = 0.759 and 0.836) at 2.3 and 3.8 μg/disk.

**Figure 8:**
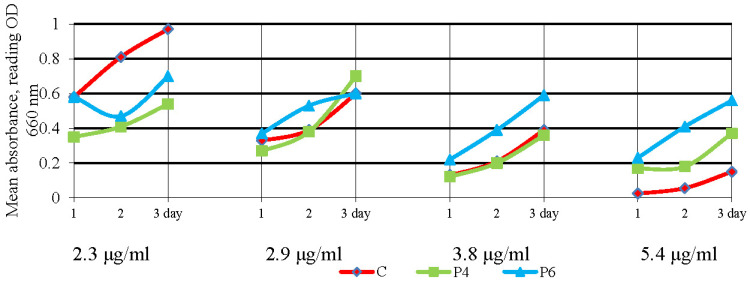
Growth of *Staphylococcus aureus* (*S. aureus*) under the action of oxytetracycline in complex with polyphosphate ester-type transporters during cultivation in a liquid medium for 3 days. Note. In this and the following figures: C—control (traditional form of antibiotic); P4 and P6 are oxytetracycline in complex with polyphosphate ester-type transporters.

Oxytetracycline + P6 was similarly effective on *S. aureus* in liquid media at a dose of 2.3 μg/mL and inhibited the growth of *S. aureus* by 27.3 to 44.2% during 3 days of cultivation, compared to the control (Figure 8).

Oxytetracycline + P4 also provided growth inhibitory effects at doses of 2.9 and 3.8 μg/mL. The highest dose, 5.4 μg/mL of oxytetracycline in complex with a polyphosphate ester-type transporter, decreases its effect on *S. aureus* growth.

**Figure 9:**
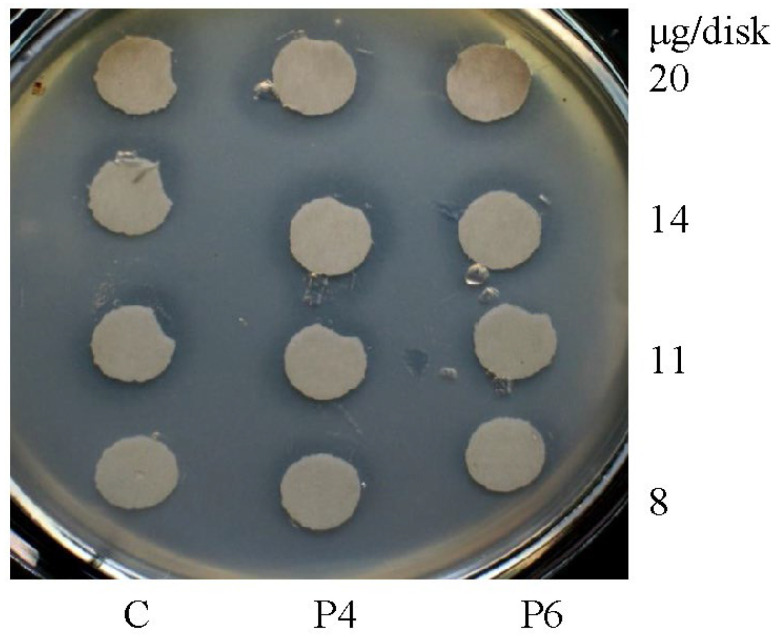
Growth inhibition of *Escherichia coli*.

The addition of the polyphosphate ester oxytetracycline complex also increased the susceptibility of *E. coli* to oxytetracycline. Inhibition of *E. coli* is most pronounced at the minimum dose (8 μg/disk) of both oxytetracycline + P4 and oxytetracycline + P6 (Figure 9, Table 2). Inhibition of *E. coli* is 29.1 to 40.8% (*p* < 0.05 to 0.001) greater than in the control (Figure 9; Table 2).

The 14 μg/disk of oxytetracycline + P4 caused a 33.0% stronger inhibition than in control. The use of 14 μg/disk oxytetracycline + P6 does not differ (1.84 ± 0.135 cm^2^) from the control. The maximum strength of influence (η^2^ = 0.695) on the growth of *E. coli* in solid medium is observed at low doses (8 μg/disk) of oxytetracycline in combination with both of the polyphosphate ester-type transporters: P4 and P6.

**Figure 10:**
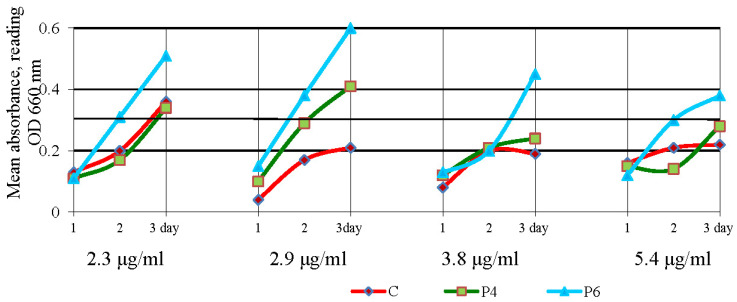
Growth of *Escherichia coli* (*E. coli*) under the action of oxytetracycline in complex with polyphosphate ester-type transporters during cultivation in a liquid medium for three days.

In liquid media, 2.3 μg/mL of oxytetracycline + P4 complex lowered *E. coli* growth by 3.7 to 19.1% compared to the control (Figure 10).

The maximum inhibition of *E. coli* was found on day 1 (19.1%), with growth decreasing on the second (13.3%) and third (3.7%) days of cultivation. Inhibition of *E. coli* by oxytetracycline + P6 was detected only on the first day of cultivation (14.6%) at a dose of 2.3 μg/mL. Therefore, the low concentration of oxytetracycline + P4 (8 μg/disk and 2.3 μg/mL) provides maximum growth inhibition of *E. coli*, compared to oxytetracycline hydrochloride.

**Figure 11:**
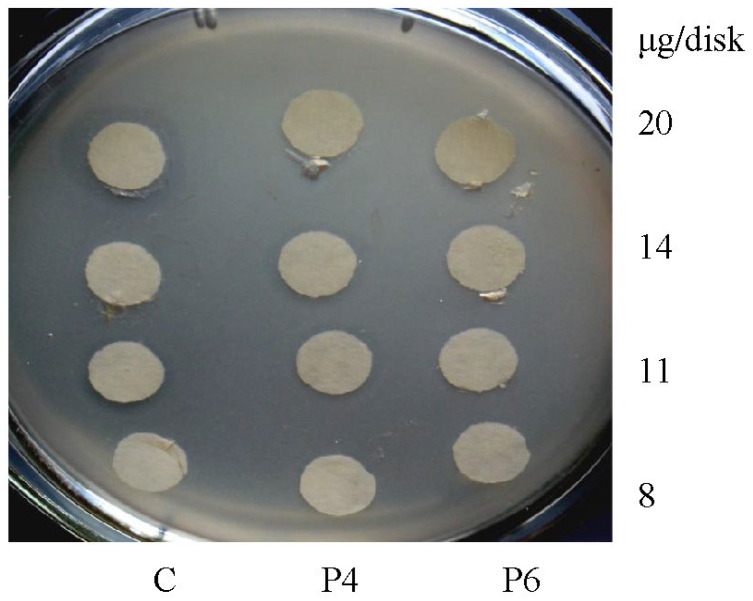
Growth inhibition of *Pseudomonas aeruginosa*.

Inhibition of *P. aeruginosa* growth during cultivation in solid medium was observed only at low doses of antibiotics in the composition of polyphosphate ester-type transporters: 8 μg/disk (Figure 11; Table 3).

The difference between the control and test samples was 5.8 to 10.8% (*p* ˂ 0.05). At higher doses of oxytetracycline (more than 8 μg/disk), the growth of the microorganism was more effectively inhibited by oxytetracycline hydrochloride than by oxytetracycline in complex with polyphosphate ester-type transporters.

**Figure 12:**
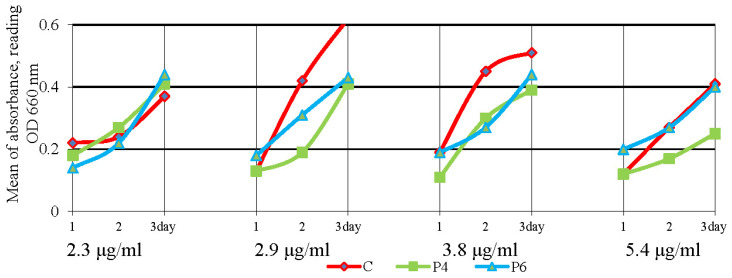
Growth of *Pseudomonas aeruginosa* (*P. aeruginosa*) under the action of oxytetracycline in complex with polyphosphate ester-type transporters during cultivation in a liquid medium for three days.

Changes in the growth of *P. aeruginosa* were found during its cultivation in a liquid medium in the presence of oxytetracycline coupled to the polyphosphate ester-type transporters. At a dose of 2.3 μg/mL of antibiotic with polyphosphate ester-type transporters, the growth of the microorganism was inhibited by 18.0 to 38.6% on the first day of cultivation, while growth was similar to controls on the second and third days (Figure 12).

Oxytetracycline + P4 inhibited the growth of *P. aeruginosa* by 33.2 to 53.9% at doses of 2.9 μg/mL, 3.8 μg/mL, and 5.4 μg/mL of oxytetracycline in the media. Oxytetracycline + P6 inhibited the growth of *P. aeruginosa* by 25.5 and 31.1% at doses of 2.9 μg/mL and 3.8 μg/mL (Figure 12).

## 4. Discussion

As shown in the results, newly created transporters of the polyphosphate ester type were able to bind and solubilize oxytetracycline. Studies have shown that polymers based on pseudo-polyamino acids, which are similar to polyphosphate ester-type nano transporters, penetrate cells and mitochondria [27,28,29]. Low-charged and amphiphilic properties facilitate membrane penetration by these compounds [30,31]. After penetrating cells and their organelles, nano transporters are destroyed by enzymes and other intracellular compounds, while amino acids (glutamic acid) are used in metabolic processes [32]. We found that cell cultures of opportunistic pathogens respond to oxytetracycline complexed with our polyphosphate ester-type transporters. Oxytetracycline polyphosphate ester-type transporters improved growth inhibition by 27.0 to 43.2% for *S. aureus* and by 29.0 to 40.8% for *E. coli*.

According to the literature, the use of oxytetracycline to inhibit the growth of *P. aeruginosa* is ambiguous. On the one hand, authors point to the insensitivity (resistance) of the microorganism to this antibiotic [33]. However, a high level of sensitivity of the microflora (75.5%) isolated from calves with bronchopneumonia has been demonstrated [34]. Our results showed that the inhibition of the growth of *P. aeruginosa* in liquid media is greater in the presence of oxytetracycline coupled to the polyphosphate transporters, as compared to oxytetracycline hydrochloride. *P. aeruginosa* was more affected by polymeric complexes with oxytetracycline compared to oxytetracycline hydrochloride on the second and third days of growth. We suggest that polyphosphate ester-type nano transporters may inhibit bacterial biofilm formation [8,10] similar to the action of known inhibitors [35]. Polyphosphate ester-type transporters can increase antibiotic permeability into cells of various species of microorganisms based on results obtained in this study. The efficiency of the polyphosphate ester-type complexes synthesized with oxytetracycline in this study is higher than that of oxytetracycline hydrochloride in low doses.

## 5. Conclusions

This study found that oxytetracycline in the complex of polyphosphate ester-type transporters has a higher antimicrobial effect in low doses than commercial oxytetracycline and has the potential for the treatment of some infections. In the future, we plan to investigate the action of these complexes in vivo and their effect on the animal body.

## Figures and Tables

**Table 1:** Area of growth inhibition of *Staphylococcus aureus* (*S. aureus*).

Oxytetracycline, μg/Disk	Area of Growth Inhibition, cm^2^			
	Control (Oxytetracycline, Hydrochloride)	Oxytetracycline + P4	Oxytetracycline + P6	η^2^
5.4	3.00 ± 0.25	3.60 ± 0.18	3.33 ± 0.24	0.232
3.8	2.90 ± 0.12	2.95 ± 0.12	3.28 ± 0.21	0.759
2.9	1.88 ± 0.17	2.78 ± 0.16 ***	1.90 ± 0.06	0.776
2.3	1.58 ± 0.07	2.40 ± 0.11 **	1.58 ± 0.06	0.836

Note. In this table, the difference is statistically significant and compared to the control **—*p* < 0.01; ***—*p* < 0.001.

**Table 2:** Area of growth inhibition of *Escherichia coli*.

Oxytetracycline, μg/Disk	Area of Growth Inhibition, cm^2^			
	Control (Oxytetracycline, Hydrochloride)	Oxytetracycline + P4	Oxytetracycline + P6	η^2^
20	2.23 ± 0.216	2.39 ± 0.125	1.90 ± 0.093	0.369
14	1.85 ± 0.102	2.46 ± 0.163	1.84 ± 0.135	0.605
11	1.76 ± 0.109	1.75 ± 0.051	1.46 ± 0.031	0.559
8	1.20 ± 0.036	1.69 ± 0.128 *	1.55 ± 0.026 ***	0.695

Note. In this table, the difference is statistically significant and compared to the control *—*p* < 0.05; ***—*p* < 0.001.

**Table 3:** Area of growth inhibition of *Pseudomonas aeruginosa*.

Oxytetracycline, μg/Disk	Area of Growth Inhibition, cm^2^			
	Control (Oxytetracycline, Hydrochloride)	Oxytetracycline + P4	Oxytetracycline + P6	η^2^
20	3.10 ± 0.12	2.10 ± 0.12	1.80 ± 0.05	0.903
14	2.63 ± 0.31	1.87 ± 0.09	1.67 ± 0.12	0.587
11	1.97 ± 0.14	1.73 ± 0.09	1.60 ± 0.08	0.383
8	1.20 ± 0.05	1.33 ± 0.07	1.27 ± 0.07	0.190

## Data Availability

Data available within the article and its Supplementary Materials.

## Supplementary Materials

The following supporting information can be downloaded at: https://www.mdpi.com/article/10.3390/antibiotics12030616/s1, Figure S1: The 1H NMR spectrum of the newly synthesized phosphorus-containing polyesters (PPE).
