# Supplementary material for: Polyphosphate Ester-Type Transporters Improve Antimicrobial Properties of Oxytetracycline
Source: Antibiotics (Basel). 2023 Mar 20;12(3):616. doi: 10.3390/antibiotics12030616 (PMC10045294; doi:10.3390/antibiotics12030616)
Supplement: Supplementary file 1 [file antibiotics-12-00616-s001.zip › antibiotics-2263196-supplementary.pdf]

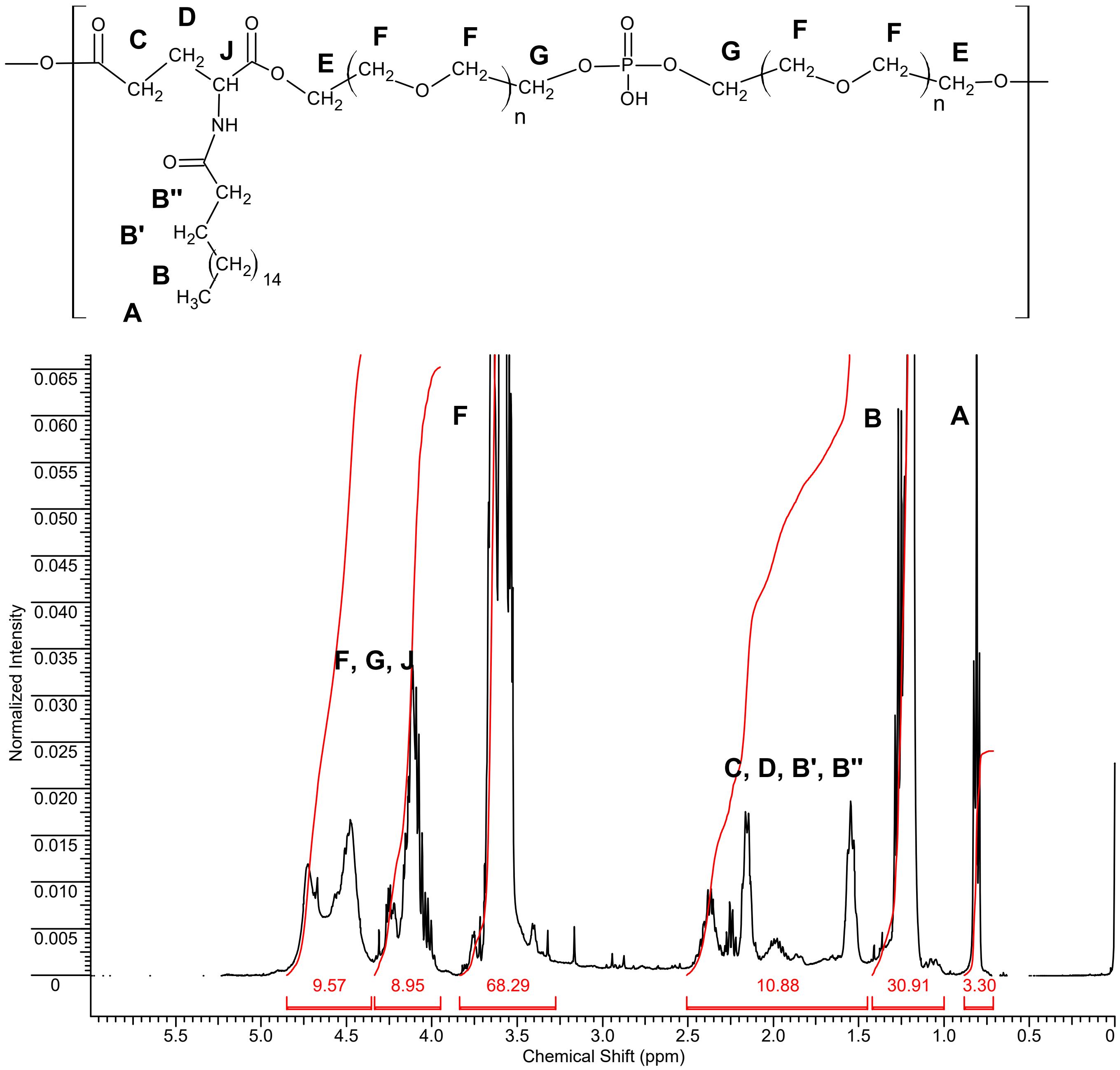

Figure S1. The ^{1}H NMR spectrum of the newly synthesized phosphorus-containing polyesters (PPE).
